# An academic achievements visualization research in the past 30 years: research on rehabilitation for head and neck cancer

**DOI:** 10.3389/fonc.2024.1389806

**Published:** 2024-06-04

**Authors:** Bo Zhou, Dian Li, Jingyi Cheng, Kexin Deng

**Affiliations:** ^1^ Department of Head and Neck Surgery, Hunan Cancer Hospital and the Affiliated Cancer Hospital of Xiangya School of Medicine, Central South University, Changsha, Hunan, China; ^2^ Department of Epidemiology, University of California, Los Angeles (UCLA) Fielding School of Public Health, Los Angeles, CA, United States; ^3^ Xiangya Stomatological Hospital & Xiangya School of Stomatology, Central South University, Changsha, Hunan, China; ^4^ Hunan Key Laboratory of Oral Health Research & Hunan Clinical Research Center of Oral Major Diseases and Oral Health & Academician Workstation for Oral-maxillofacial and Regenerative Medicine, Central South University, Changsha, Hunan, China; ^5^ Department of Plastic and Reconstruction, The Third Xiangya Hospital of Central South University, Changsha, Hunan, China

**Keywords:** head and neck cancer, academic visualization, rehabilitation, bibliometrics, CiteSpace

## Abstract

**Background:**

Head and neck cancer acts as the sixth most common malignant tumor worldwide with an increasing incidence. The needs and methods of its rehabilitation are diverse and constantly evolving.

**Objective:**

This study aims to provide an in-depth depiction and visualization of the knowledge structure, hotspots, and emerging trends within the domain in the past 30 years through utilizing bibliometric analysis.

**Methods:**

The literature about rehabilitation for head and neck cancer in Web of Science was collected. CiteSpace and VOSviewer were used to analyze main countries, institutions, authors, journals, subject hotspots, trends, frontiers, etc.

**Results:**

A total of 1869 papers have been published since 1994. These publications were written by 874 authors from 514 institutions in 74 countries. The United States published 397 papers in this field and ranked first. Head & Neck is the most widely published journal, with Finizia, Caterina as the core author. The main keyword clustering includes terms such as #0 mandibular reconstruction (2009); #1 functional impairment (2014); #2 device lifetime (2006); #3 head and neck cancer (2003); #4 maxillofacial prosthetics (2004); #5 squamous cell carcinoma (2002); #6 readiness for return to work (2009); #7 total laryngopharyngectomy (2004). The current research frontier that has been sustained is “survivors”, “reliability”, and “meta analysis”.

**Conclusion:**

We reveal the current status, hotspots, and trends in the field of rehabilitation for head and neck cancer. And we provided new academic insights into the characteristics and limitations of the field’s development.

## 1 Introduction

Head and neck cancers (HNCs) refer to a heterogeneous group of malignancies occurring from the upper aerodigestive tract (such as the oral cavity, oropharynx, larynx, nasopharynx, and hypopharynx) (1). The predominate histological subtype of HNCs is squamous cell carcinoma (accounting for over 90%) (2). According to the GLOBOCAN database (3, 4), HNCs are currently the sixth most common malignancy worldwide, with an estimated 930,000 new cases and 470,000 deaths per year, and the incidence is expected to continue to rise. Clinical symptoms of HNCs include a lump in the neck, a non-healing sore in the mouth or throat, swallowing difficulties, hoarseness or other voice changes (5). Common risk factors for developing HNCs include tobacco use, alcohol consumption, and human papillomavirus (HPV) infection (6, 7).

Currently, while the incidence of HNC is rising, survival rates have also improved, particularly among patients with HPV-related oropharyngeal cancers (8). HNCs can be treated in a variety of ways, including surgery, radiotherapy, chemotherapy, targeted therapy, immunotherapy, etc. Despite the constant advancement of new strategies such as immunotherapy and targeted therapy, surgery combined with adjuvant radiotherapy or chemotherapy (according to stage, pathologic feature, etc.) still dominates the current treatment paradigms for HNCs (9). Nevertheless, long-term impairment, disability, and handicap in HNC patients are presented because of toxicity from disease itself and treatments (especially surgical resection and chemoradiotheraphy) (10). Therefore, it is essential to focus on the rehabilitation of patients with HNC to cope with the overwhelming physical and functional changes.

Rehabilitation intervention plays an extremely important role in improving function and helping patients achieve a satisfactory quality of life. The changes in the physiological structure of head and neck vary with the location and size of the primary lesion, and the adopted treatment methods. Thus, patients with HNC have multiple rehabilitation needs, and rehabilitation interventions are patient-specific, aimed at preventing, restoring, compensating, and alleviating symptoms and sequelae of treatment for optimal functioning. In the rehabilitation of patients with HNC, various issues need to be addressed, including difficulties in mouth opening, chewing, swallowing, voice/speech, airway obstruction, lymphedema, neck and shoulder dysfunction, and pain control (11, 12). There are various strategies for rehabilitation, including exercise training, behavioral therapy, psychological intervention, prostheses, instrument assistance, etc. The rehabilitation of patients with HNC is moving towards an integrated and coordinated direction. Based on interdisciplinary clinical and community resources, new rehabilitation strategies and models are explored in order to ultimately achieve individualized rehabilitation goals (13). The field of rehabilitation for HNC is on the rise, and we need to systematically review the research achievements in this field to better determine the direction of development.

Bibliometrics arose in the early 20th century. In 1969, Pritchard announced that bibliometrics had become an independent discipline (14) and was widely applied in scientific literature analysis (15). Bibliometric analysis is a method of quantitatively investigating, examining and analyzing the research results in a specific field (16). The literature analysis mainly aims to get detailed information, including authors, keywords, references, institutions, countries, and so on, which can be further analyzed for property analysis and performance analysis to evaluate the development of a certain field (17). Advances in modern imagery and visual technology can help us add literature analysis more intuitively. Now, the basis and emphasis of visual analysis of bibliometrics is co-citation. If two articles are cited in one or more articles at the same time, it is considered that there is a co-citation relationship between the two articles, which can also be synonymously used by authors, journals, keywords, countries, institutions, etc., to explore and visualize the internal relationship between them (18). For instance, diverse journals have similar research direction, and various authors have similar research topic. Ma and Xi said that the best way to interpret the data is to use the co-cited visual measurement method in bibliometrics, as the results are more reliable and comprehensive (19).

This paper aims to provide readers with a comprehensive and systematic study of scientometrics in the rehabilitation of patients with head and neck cancer. Specifically speaking, the analysis of this paper focuses on some key issues such as the cooperation and co-emergence of states, institutions, authors, and so on. Special attention is also paid to the temporal and spatial changes in the development priorities of the whole field and the frontier trends. CiteSpace is a visualization tool for analyzing citations obtained in the Web of Science Core Collection (WoSCC) (20). This software can provide great support for the scientific research of skin and probiotics, and fill the knowledge gap in the bibliometrics review of this subject. The main objectives of this study are to: (1) summarize the research on rehabilitation of patients with head and neck cancer in the past 30 years under the background of globalization from the perspectives of authors, institutions, countries, etc.; (2) study the hot research topics in this field and their characteristics; (3) summarize the overall development trend and characteristics, and analyze the research direction with potential value based on trend analysis.

## 2 Methods

### 2.1 Research methods

As a quantitative analysis method, Bibliometrics takes the external characteristics of scientific documents as the research object. This study adopts mathematical and statistical methods to describe, assess and forecast the status quo and development trend of science and technology. Bibliometrics methods is helpful to study the underlying knowledge structure of academic literature, including keywords, references, and so on. Besides, these methods help to integrate and visualize the results for further study the field. Much literature data can be analyzed with bibliometric software to get visual results.

CiteSpace is an information visualization software developed by Dr. Chen Chaomei of Drexel University based on citation analysis theory, through which we can know the structure, pattern and distribution of scientific knowledge. The graph formed is called “scientific knowledge graph”, which is mainly used to sort out the theoretical perspective, evolutionary path, development trend, academic history and hot spot scanning in the field of scientific research. It is practical quantitative analysis software for document analysis. Citespace analysis methods include co-citation analysis, co-occurrence analysis, burst detection and cluster analysis. The co-citation analysis is used to study the co-citation relation between the two studies in the third study. The more frequently two studies are cited together, the more similar they are and the more related they are to each other. Co-occurrence analysis calculates the number of occurrences of a series of keywords in the literature of the investigated field and measures their affinity by co-occurrence. Burst detection can monitor changes in the use of specific keywords. Cluster analysis is to group objects on the basis of their similarity and analyze multiple generated clusters. Centrality is a key indicator of an object’s importance. Nodes with intermediate centrality greater than 0.1 are called central nodes or key nodes, which are critical and influential in the research field and often serve as Bridges connecting different research objects such as articles, keywords and countries. CiteSpace is uniquely positioned to identify key points and future trends in the field of research. As a result, the bibliometrics analysis software CiteSpace is used to analyze the existing papers on rehabilitation for head and neck cancer. Critical readings are also performed to further study the key research and provide key insights into this topic.

### 2.2 Data resource

**Figure 1:**
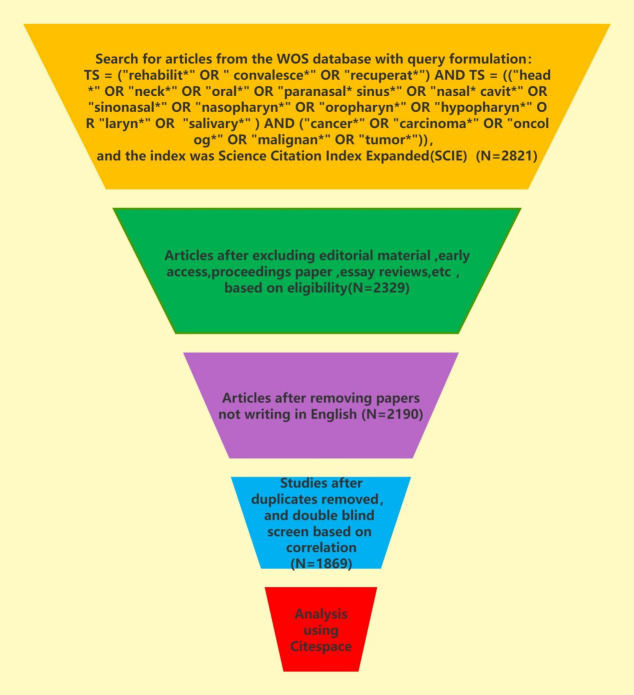
The search strategy used for the present bibliometric analysis.

This paper selects Web of Science Core Collection (WoSCC) and the index is Science Citation Index Expanded(SCIE). Retrieval formula of this paper is as follows: TS = (“rehabilit*” OR “ convalesce*” OR “recuperat*”) AND TS = ((“head*” OR “neck*” OR “oral*” OR “paranasal* sinus*” OR “nasal* cavit*” OR “sinonasal*” OR “nasopharyn*” OR “oropharyn*” OR “hypopharyn*” OR “laryn*” OR “salivary*”) AND (“cancer*” OR “carcinoma*” OR “oncolog*” OR “malignan*” OR “tumor*”)). The time span is from January 1, 1994 to December 31, 2023. A total of 2821 publications (2329 articles, 390 reviews, 47 conference abstracts and 55 others) were retrieved on January 1st, 2024. After eliminating duplicate articles, 2109 articles written in English were selected. We subsequently conducted a double-blind screening by correlation and identified the same subsets based on the following criteria: (1) The research subjects must be patients who have undergone treatment for head and neck cancer, but not other parts of the body. (2) The research subjects must be humans, not cats, dogs, pigs, or other animals. (3) The main focus of the research must be rehabilitation treatment. (4) According to the National Cancer Institute (NCI), head and neck cancers (HNCs) comprise a diverse group of malignancies that develop in or around the oral cavity, pharynx (throat), larynx (voice box), nasal cavity, paranasal sinuses, and salivary glands (National Cancer Institute. (n.d.). Head and Neck Cancers. Retrieved from [https://www.cancer.gov/types/head-and-neck/head-neck-fact-sheet]). the research site should exclude the eyes, thyroid, the brain and its surrounding tissues and so on. At last, this paper made a bibliometric analysis of 1869 articles. The literature retrieval and screening process is recorded in **Figure 1** and **Table 1**.

**Table 1:** The search strategy used for the present bibliometric analysis.

Category	Specific Standard Requirements
Research database	Web of science core collection
Citation indexes	SCIE
Searching period	1994-01-01 to 2023-12-31
Language	“English”
Document types	“Articles”
Data extraction	Export with full records and cited references in plain text format
Query formulation	TS = (“rehabilit*” OR “ convalesce*” OR “recuperat*”) AND TS = ((“head*” OR “neck*” OR “oral*” OR “paranasal* sinus*” OR “nasal* cavit*” OR “sinonasal*” OR “nasopharyn*” OR “oropharyn*” OR “hypopharyn*” OR “laryn*” OR “salivary*”) AND (“cancer*” OR “carcinoma*” OR “oncolog*” OR “malignan*” OR “tumor*”)).
Sample size	1869

## 3 Results

### 3.1 Analysis of publishing trend

**Figure 2:**
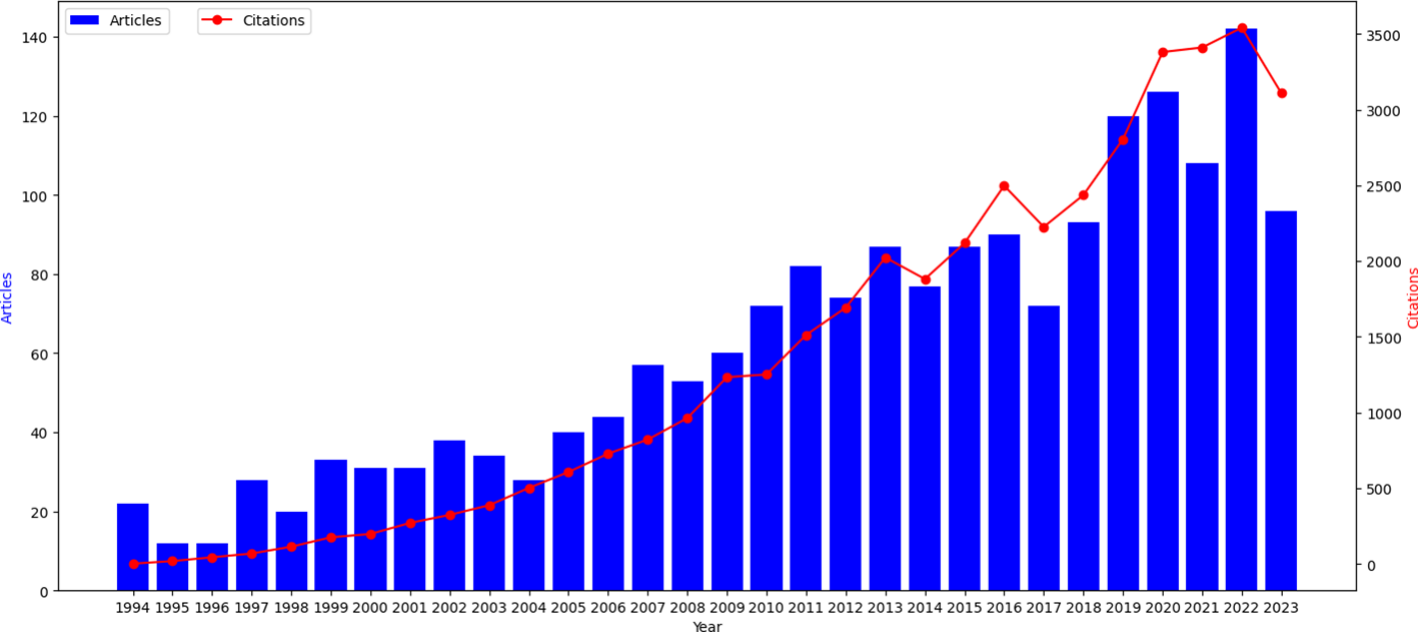
Time evolution of the total number of publications and citations in the WOS database.

Publication and citation trends are important indicators to measure the research and development of certain scientific areas. Therefore, by drawing the literature quantity-time curve, we can effectively evaluate the current research status in the field and further predict its development trends. **Figure 2** shows the annual distribution of articles related to head and neck cancer rehabilitation on the WOS since 1994. Overall, research in the field of head and neck cancer rehabilitation has made significant progress. The number of articles and citations maintained a stable growth rate, with an average of 62 articles published and 1,345 citations per year. In the first 15 years, the number of articles was relatively small, with only about 32 articles and an average citation of 349 per year. In the latter 15 years, the curve for article publication increased, with about 92 articles published and 2,341 citations. The total number of citations also increased from less than 3 to 3,108. Although there is a slight decline in research numbers in 2023, research on head and neck cancer rehabilitation treatment is rapidly developing. Based on this trend it is expected that research in this field will increase in the coming years.

### 3.2 National, institutional, author and journal analysis

#### 3.2.1 National analysis

**Figure 3:**
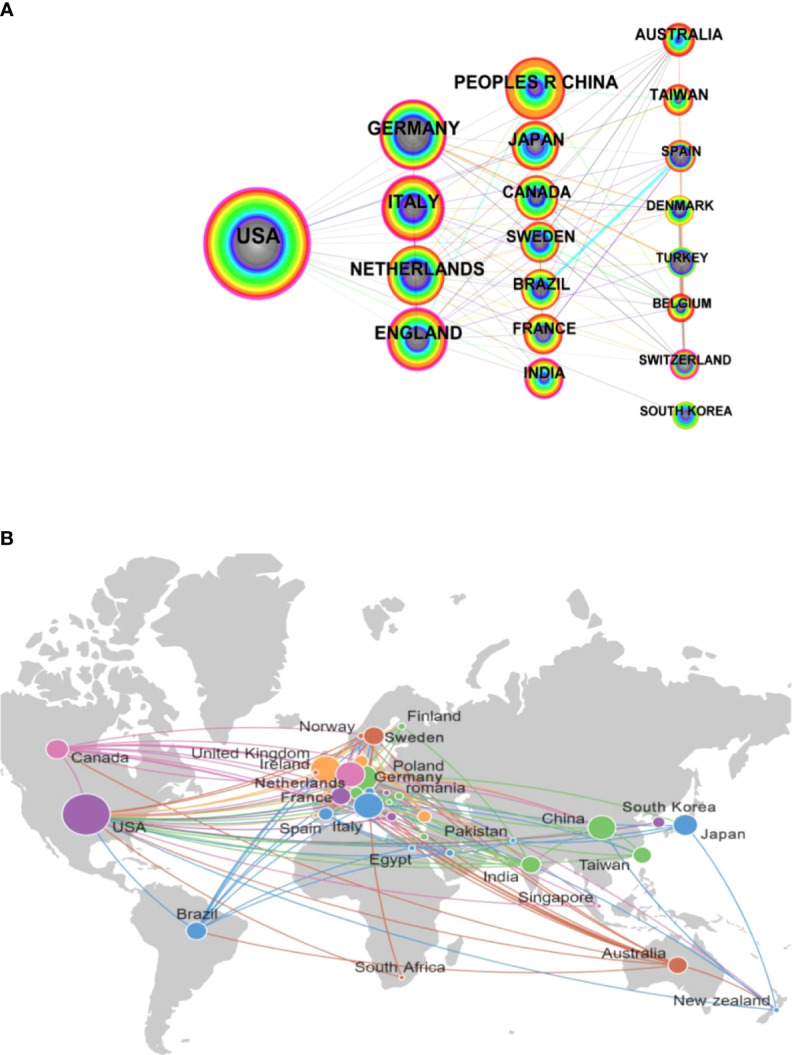
Analysis of publications among countries. **(A)** Cooperation network of countries. **(B)** Analysis of geographical distribution of countries.

Through the quantitative visual analysis of national cooperation, it can not only reflect the academic exchanges and cooperation of countries in this field, but also identify the core countries of research. In this study, “country” was selected as the analysis object in CiteSpace with a threshold value of the top “20”, “time slice” time of “1994-2023”, and “per slice year” of “1”. Finally, a national analysis map with 74 network nodes and 264 connections with a density of 0.0977 was obtained, as shown in **Figure 3**. Countries with purple outer rings indicates their intermediate centrality > 0.1, and the thickness of the purple ring indicates the degree of intermediate centrality, reflecting the importance of their position in the network. **Table 2** lists the top 20 countries. As shown in **Table 2**, frequency represents the number of publications, while centrality represents the importance of one country’s position in this field. From **Figure 3**, it could be seen that the more international relations a country has, the higher its centrality and the greater its power.

**Table 2:** The Centrality and count of literature in countries.

Country	Year	Centrality	Frequency
USA	1994	0.54	397
GERMANY	1996	0.14	161
ITALY	1994	0.14	149
NETHERLANDS	1994	0.08	148
UNITED KINGDOM	1994	0.24	138
PEOPLES R CHINA	1994	0.02	134
JAPAN	1995	0.01	104
CANADA	2000	0.09	88
SWEDEN	1994	0.07	75
BRAZIL	2000	0.01	73
FRANCE	1998	0.04	69
INDIA	1995	0.12	65
AUSTRALIA	1995	0.07	64
TAIWAN	1997	0	60
SPAIN	1996	0.01	37
DENMARK	1994	0	35
TURKEY	2001	0	33
BELGIUM	1997	0.02	32
SWITZERLAND	1999	0.11	31
SOUTH KOREA	1998	0	27

As shown in **Table 2**, the United States has published 397 articles with the highest centrality of 0.54, indicating a significant impact on the field as measured by bibliometric indicators. In addition, the top five countries in terms of publication volume are the USA, Germany, Italy, Netherlands, and the United Kingdom. Among these countries, the centrality of the USA, Germany, Italy, and the United Kingdom is greater than 0.1, indicating that these countries have achieved extensive transnational cooperation in this field and are pivotal in bridging information flows, as evidenced by their high betweenness centrality. The publication volume of Peoples R China ranked sixth, with 134 related studies, but its centrality was relatively low, indicating that the international communication in the research process of Peoples R China was still insufficient. In addition, research outputs from India and Switzerland, with centrality scores above 0.1, play a critical role in connecting various international research clusters. These scores reflect their proactive engagement in international cooperation, despite their relatively lower publication volumes. Based on **Figure 3** and **Table 2**, it is clear that there is a concentration of research activities in developed countries, particularly in Europe and North America. This geographic distribution highlights their active involvement and a well-established infrastructure that supports extensive international research collaborations. Additionally, the centrality data shows that, apart from India, countries with a centrality score greater than 0.1 are mainly developed countries. This high centrality indicates their central roles in global research networks, facilitating collaborative and multi-centered scientific inquiries. These countries often lead the initiative and management of large-scale research projects involving multiple countries and institutions. In terms of time, countries such a sthe USA, Italy, Netherlands, United Kingdom, Peoples R China, Swden, Denmark started the earliest research and published a large amount, which has a foundational role in the field.

#### 3.2.2 Institutional analysis

**Figure 4:**
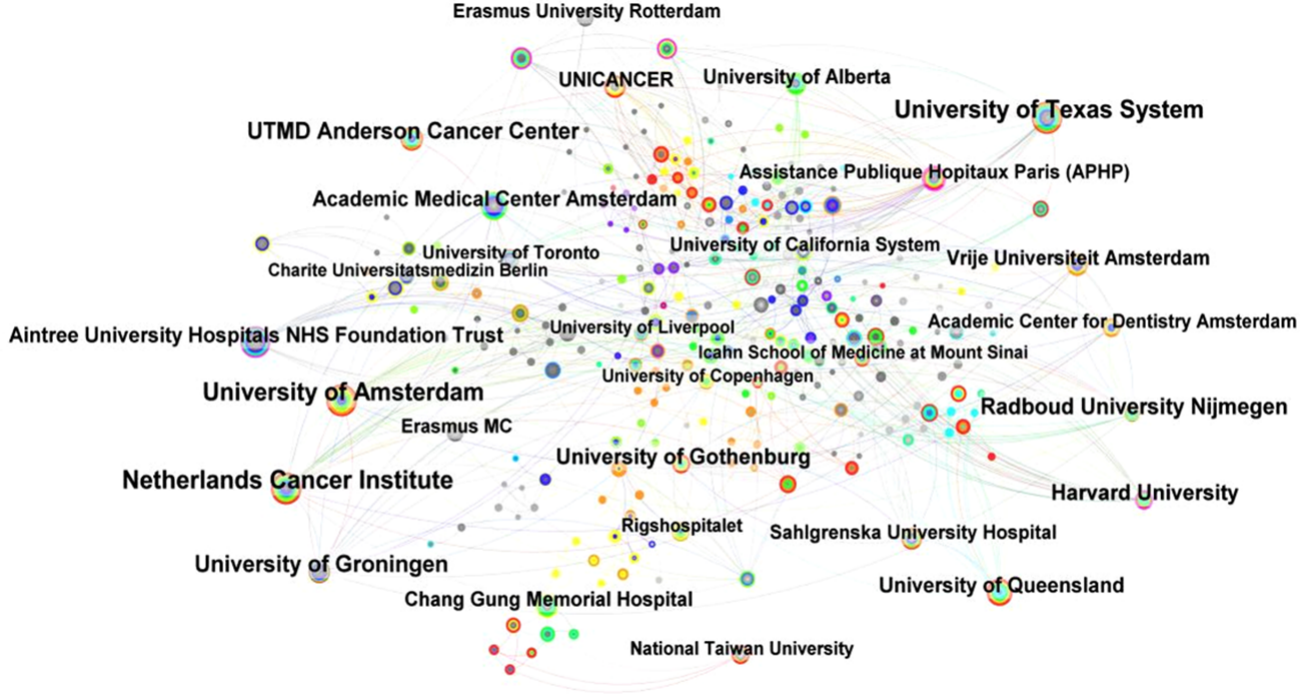
Interinstitutional cooperation network.

Taking the “institution” in CiteSpace as the analysis object, a cooperation analysis map with a density of 0.0071 is obtained, including 514 network nodes and 942 connections (**Figure 4**). The nodes in **Figure 4** are relatively dense, indicating that interinstitutional cooperation in the field of head and neck cancer rehabilitation is relatively frequent, and most research institutions have collaborated. Some of the regional features of institutional cooperation indicate the need to maintain research cooperation between institutions to promote academic exchanges in the field of head and neck cancer rehabilitation.

At the same time, this paper collated the top ten institutions in terms of publication volume of literature related to head and neck cancer rehabilitation in **Table 3**. As shown in **Table 3**, the Netherlands Cancer Institute, the University of Texas System and the University of Amsterdam ranked first to third, with the publication volumes of 48, 44, and 42 respectively. However, their centrality is very low, indicating that they are primarily used to conduct independent research with less collaboration with other institutions. On the contrary, institutions with centrality greater than 0.1, such as Harvard University, Aintree University Hospitals NHS Foundation Trust and others, have published relatively little literature. Additionally, three other institutions with intermediary centrality greater than 0.1 (not listed in the table), such as Assistance Publique Hopitaux Paris (APHP), University of London, and State University System of Florida have publication volumes of 18, 12, and 10 respectively. Although these institutions produce fewer publications, they are more inclined to collaborate on research and act as a bridge between many high-level research institutions. In addition, the Netherlands Cancer Institute, the University of Groningen, and Harvard University are the earliest institutions to carry out research on head and neck cancer rehabilitation treatment, laying the foundation for the development of the field.

**Table 3:** The top 10 institutions of publication.

Institution	Year	Centrality	Frequency
Netherlands Cancer Institute	1994	0.02	48
University of Texas System	1997	0.09	44
University of Amsterdam	2002	0.06	42
UTMD Anderson Cancer Center	1997	0.02	33
University of Groningen	1994	0.02	32
Radboud University Nijmegen	1997	0.01	28
Harvard University	1994	0.13	26
University of Gothenburg	2001	0.06	26
Academic Medical Center Amsterdam	1998	0.01	24
Aintree University Hospitals NHS Foundation Trust	2004	0.12	23

#### 3.2.3 Author cooperation analysis

**Figure 5:**
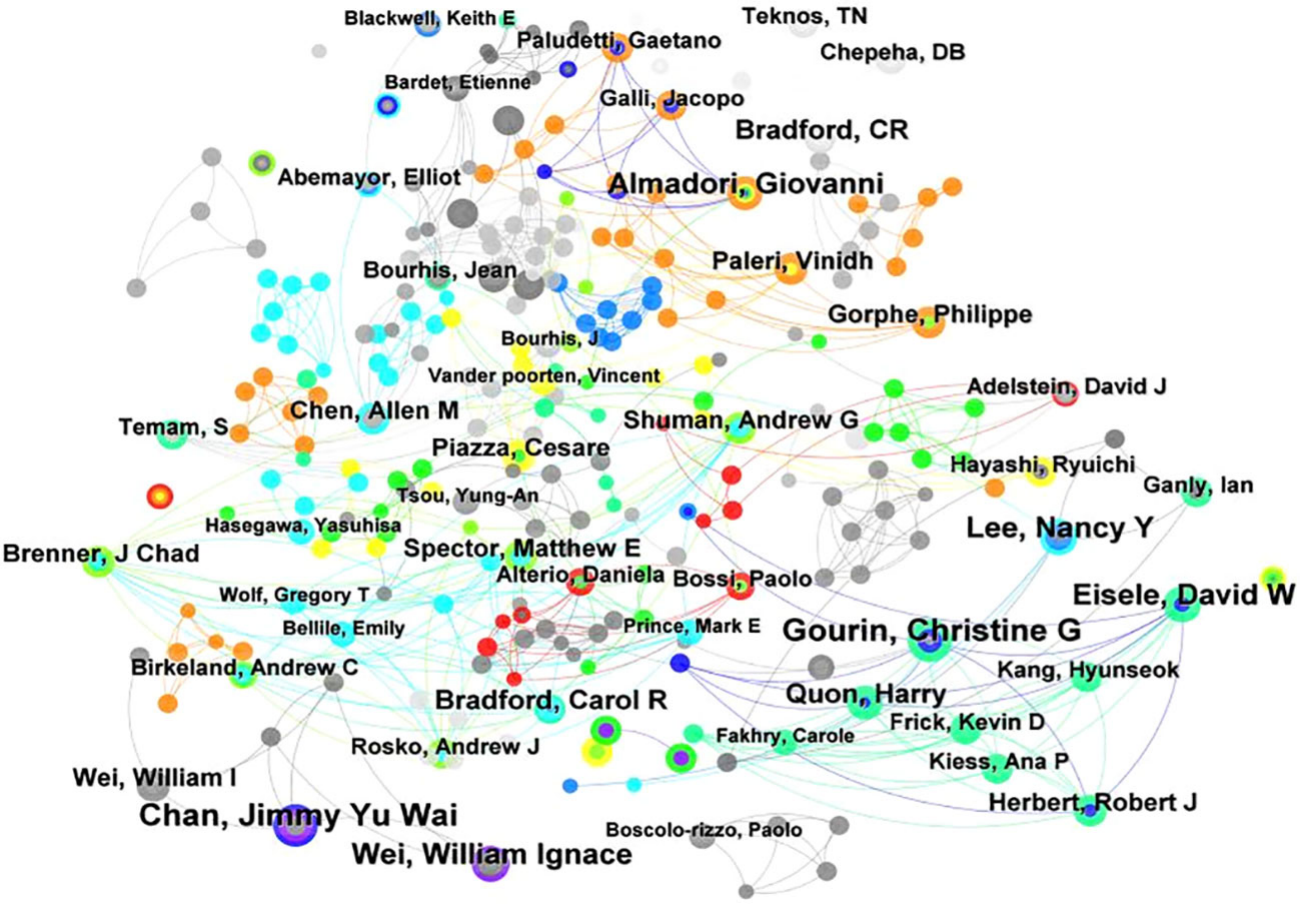
Analysis diagram of author collaboration network.

Based on the analysis of “authors” in CiteSpace, we utilized the Co-author Count method in our analysis; an author analysis map with 874 network nodes, 895 connections, and a density of 0.0023 was obtained (**Figure 5**; **Table 4**). In this map, the three largest nodes are Finizia, Caterina; van der Molen, Lisette; and van den Brekel, Michiel W M, with publication counts of 17, 15, and 13, respectively. This indicates that they are the most prolific contributors in the field. Van der Molen, Lisette and van den Brekel, Michiel W M are not only the most productive scholars, but they are also two scholars who work very closely with each other. At the same time, Lisette van der Molen’s research has been notably active and impactful in recent years, as evidenced by her high publication frequency and significant citation burst starting in 2020, and there is little connection between them and Finizia, Caterina. In addition, the research of Hilgers, FJM is relatively old, indicating that he is the founder of the field. Moreover, as can be seen from the author collaboration, close academic cooperation is conducive to the production of scientific research achievements.

**Table 4:** Top 10 authors in publication count.

Frequency	Burst Degree	Beginning	Ending	Author
17	3.87	2012	2017	Finizia, Caterina
15	4.54	2020	2023	van der molen, Lisette
13	0	None	None	van den brekel, Michiel W M
11	0	None	None	Tuomi, Lisa
10	4.71	2011	2016	Hilgers, Frans J M
8	4.41	2009	2012	Tschiesner, Uta
8	4.44	2017	2019	Ward, Elizabeth C
8	0	None	None	Ackerstaff, AH
8	4.09	1999	2005	Hilgers, FJM
7	3.69	2019	2021	Wessel, Irene

Beginning’ and ‘ending’ years indicate the start and end of a period during which an author’s work experienced a notable peak in citations, as measured by the burst degree. This period highlights when the research was most recognized and does not necessarily correspond to the first or last publications of the authors.

#### 3.2.4 Journal co-citation analysis

**Figure 6:**
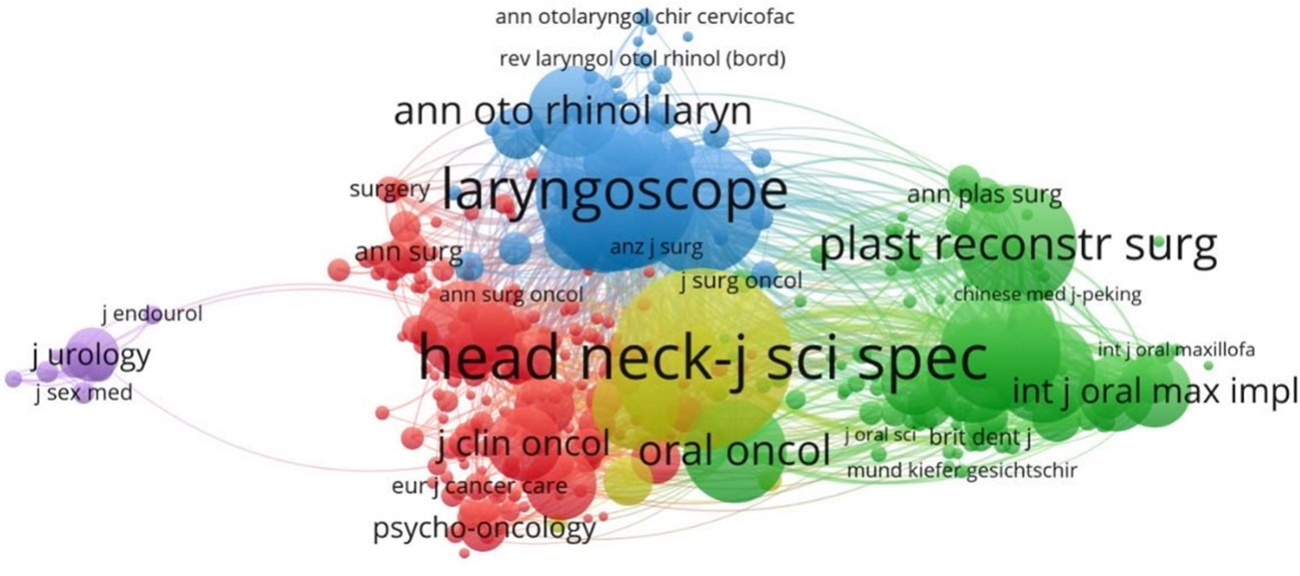
Analysis diagram of journal co-citation network.

By using VOSvier to perform a statistical analysis of publications and journal citations (**Figure 6**), we identified three core journals that are the most authoritative in the field. By observing the node size in **Figure 6** and **Table 4**, we can intuitively see the journals that publish the most articles in this field. **Table 5** lists the top 10 journals by citations in the field. From the comparison of journal publications, citations or impact factors, the core journals of rehabilitation in head and neck cancer are *Head And Neck-Journal For The Sciences And Specialties Of The Head And Neck*, *Archives of Otolaryngology- Head & Neck Surgery*, *Laryngoscope. Head And Neck-Journal For The Sciences And Specialties Of The Head And Neck* has published the highest number of articles and achieved the highest citations, making it an undeniable core journal. The journal with the highest average citation per article is Cancer. Although Cancer has only eight articles, its published articles have an important position and profound academic significance in the field.

**Table 5:** Journals with the Top10 citation numbers.

	Journal	Counts	Citations
1	Head And Neck-Journal For The Sciences And Specialties Of The Head And Neck	90	2916
2	Archives of Otolaryngology- Head & Neck Surgery	36	2275
3	Laryngoscope	58	1831
4	Journal Of Oral And Maxillofacial Surgery	58	1537
5	European Archives Of Oto-rhino-laryngology	60	1232
6	Supportive Care In Cancer	48	1094
7	Oral Oncology	46	964
8	Cancer	8	894
9	Clinical Oral Implants Research	16	856
10	International Journal of Oral and Maxillofacial Surgery	28	805

### 3.3 Keywords cluster and co-citation analysis, frontier and trend analysis

#### 3.3.1 Keywords cluster and co-citation analysis

**Figure 7:**
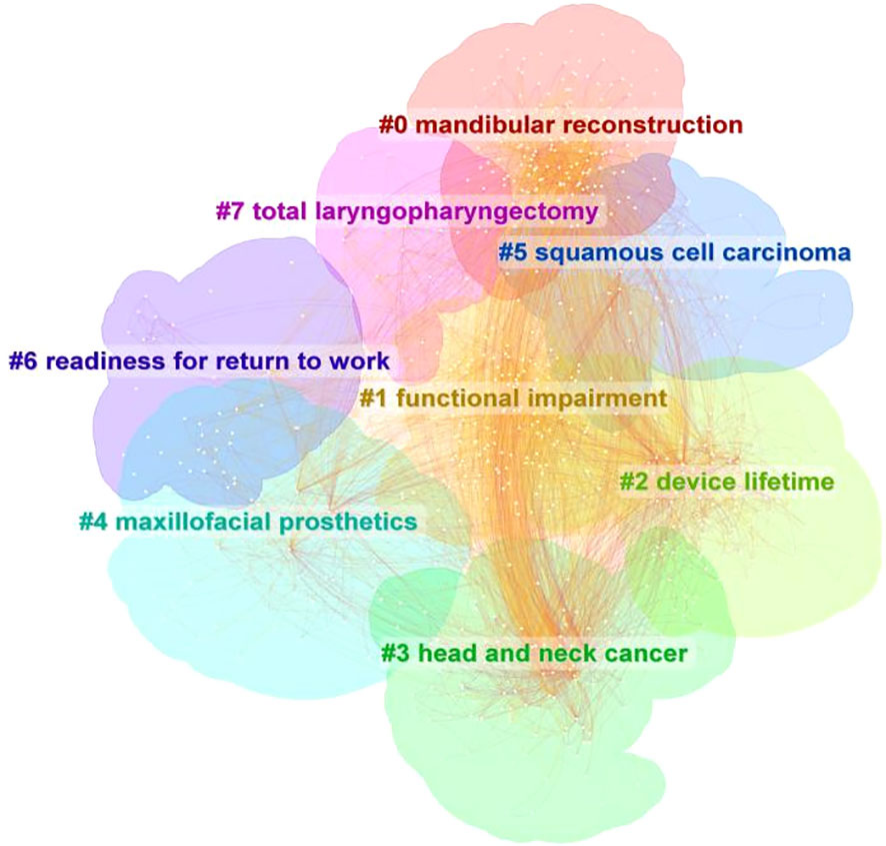
Analysis of keywords clustering.

In this paper, Citespace is used to cluster keywords, the “cluster” option is selected, and the pathfinder algorithm is used to cut off the connection lines to ensure the classification rationality of clustering. The results are shown in **Figure 7**, which reflects the research topics in the field of head and neck cancer rehabilitation since 1994. A total of 8 clusters are obtained.

As can be seen from **Figure 7**, this study generated a total of 8 clusters (the cluster numbering starts from 0), which were: #0 mandibular reconstruction (2009); #1 functional impairment (2014); #2 device lifetime (2006); #3 head and neck cancer (2003); #4 maxillofacial prosthetics(2004); #5 squamous cell carcinoma (2002); #6 readiness for return to work(2009); #7 total laryngopharyngectomy (2004).

In the field of rehabilitation treatment for head and neck cancer, it can be observed that research initially focused on the most representative pathological type, squamous cell carcinoma, especially highlighted in 2002. In the following year (2003), researchers began to pay more attention to broader aspects of head and neck cancer. By 2004, researchers were more interested in studying content related to cancer surgical methods and maxillofacial prosthetics, such as “total laryngopharyngectomy” and “maxillofacial prosthetics”. Studies on prosthetic devices became popular in 2006, including the types, materials, transplantation methods, functions, and lifespans of prostheses, as shown in the cluster#2 ‘device lifespan | prosthetic leakage’. Mandibular reconstruction remained a focus for researchers over time, and around 2009, studies on keywords related to reconstruction, such as dental implants, flaps, oral health-related quality, and other aspects of restoration, became more popular. In addition, the definition of rehabilitation gradually shifted from focusing solely on survival to placing more emphasis on patients’ social functions, as seen in “readiness for return to work”. More recently, research has focused on “functional impairment”, with studies on swallowing difficulties, language disorders, hearing loss, and other functional impairment receiving significant attention.

#### 3.3.2 Analysis of research frontiers

**Figure 8:**
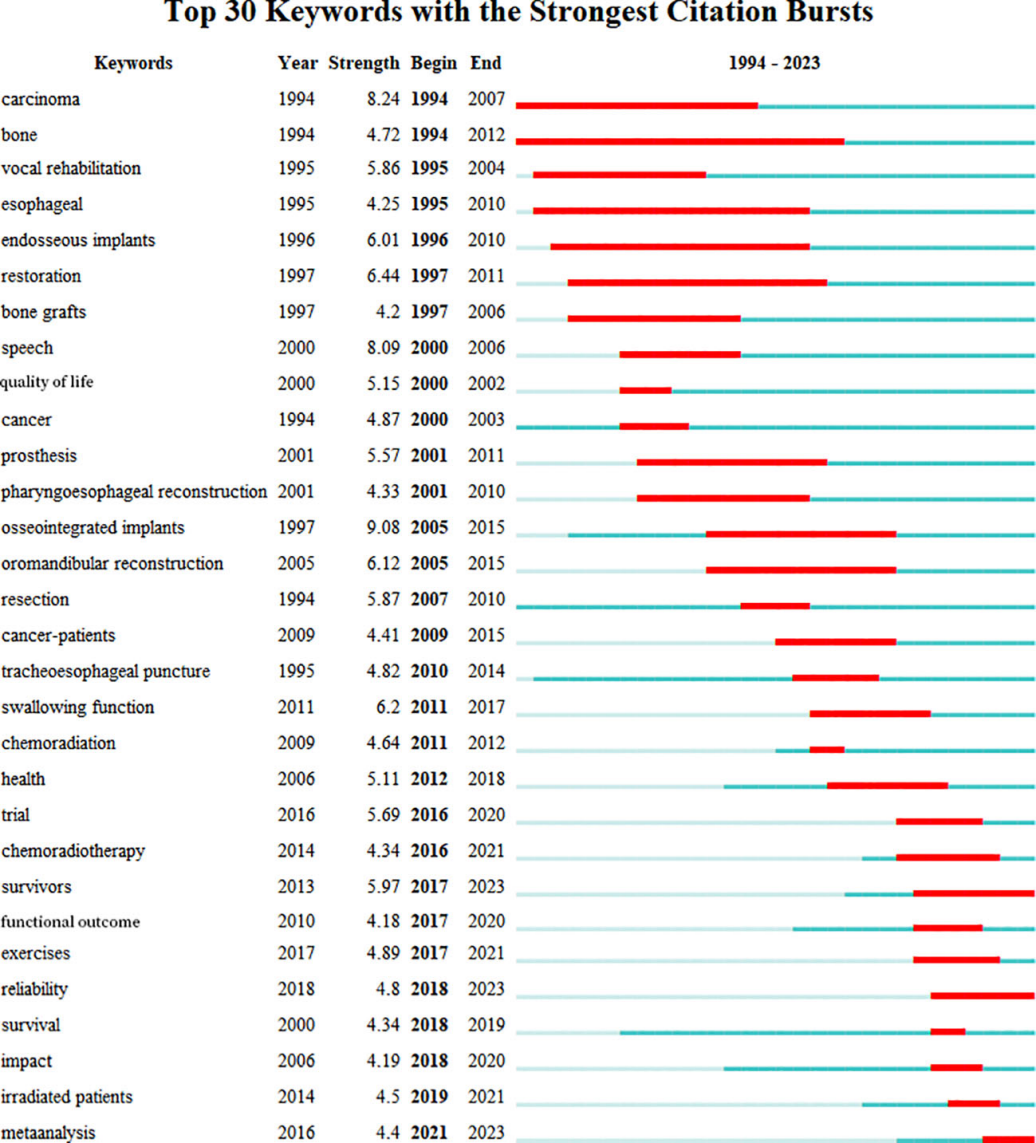
Top 30 keywords with the strongest citation bursts.

In this study, the Bursts detection algorithm of Citespace software was used to obtain the evolution map of hot topics in the field of head and neck cancer rehabilitation research on Web of Science, namely, keyword burst. As shown in **Figure 8**, this study generated the top 30 burst keywords with their burst intensity and duration in the field. The time interval is indicated by the blue line. The time period during which an outbreak appeared is displayed as a red segment, indicating the beginning year and the end year of the outbreak duration.

It can be seen that the burst keywords can be divided into two parts according to the start time, namely, before and after 2008. The former has a longer average eruption duration, about 10.6 years, while the latter has a shorter duration a higher update frequency, and a shorter eruption duration at the research frontier, about 4.9 years, indicating that the scholars in the latter part are more active, the field develops rapidly, and the changes in frontiers and key points change more frequently. From the keyword explosion chart, we can see four trends: Firstly, research has shifted from focusing solely on survival and sustainability to paying increasing attention to quality of life, health, and complete functionality, as evidenced by the evolution from keywords such as “carcinoma”, “restoration”, “quality of-life” to “health”, “exercise”, “functional outcome”, and “reliability”. Secondly, there has been increasing emphasis on multi-center research and clinical proof data, as shown by keywords such as “trial” and “meta analysis” in recent years. Thirdly, there has been a shift from simple transplantation of prostheses to innovative cancer surgery methods, as evidenced by the keywords such as “endosseous implants”, “bone grafts”, “prosthesis”, and “osseointegrated implants” to “pharyngoesophageal reconstruction” and “oromandibular reconstruction”. Fourthly, there has been increasing attention on the rehabilitation of non-surgical patients, namely radiation therapy received and chemotherapy received patients, as evidenced by keywords such as “chemoradiation” and “chemoradiotherapy” that emerged later. Ongoing research themes are “survivors”, “reliability”, and “meta analysis”.

#### 3.3.3 Trends analysis

**Figure 9:**
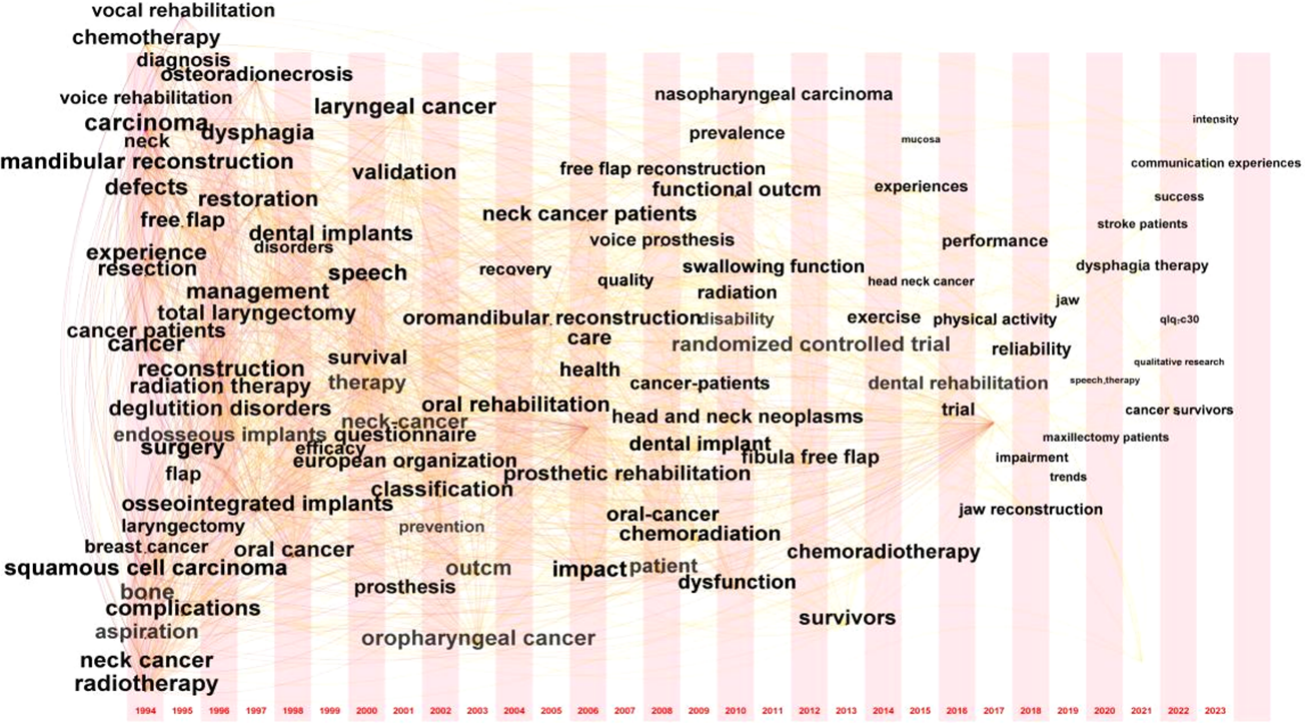
The Timezone chart of keywords in region of head and neck cancer rehabilitation.

The keywords timezone map of the domain was generated by using Citespace to select ‘Timezone’ as the analysis node, that is, the keyword changes over time (**Figure 9**). The specific indicators and thresholds are set as follows: When the time slice is ‘1’, the current graph is generated, as shown to the **Figure 9**. The time zone diagram describes the change of keywords over time. The time slice corresponding to the keywords is the year in which the keyword first appears.

We can observe that in the 1990s, the initial focus was on the treatment methods, complications, pathological types, and skin flap transplantation for head and neck cancer. In the early 21st century, there was a surge in research on post-treatment prosthetic transplantation, focusing on the quality of life and functional indicators for rehabilitation after head and neck cancer treatment. Additionally, researchers are increasingly emphasizing the importance of clinical evidence and multi-center studies. In recent years, these keywords have received continuous research attention, and the importance of exercise rehabilitation therapy has been recognized.

### 3.4 Literature co-citation analysis

**Figure 10:**
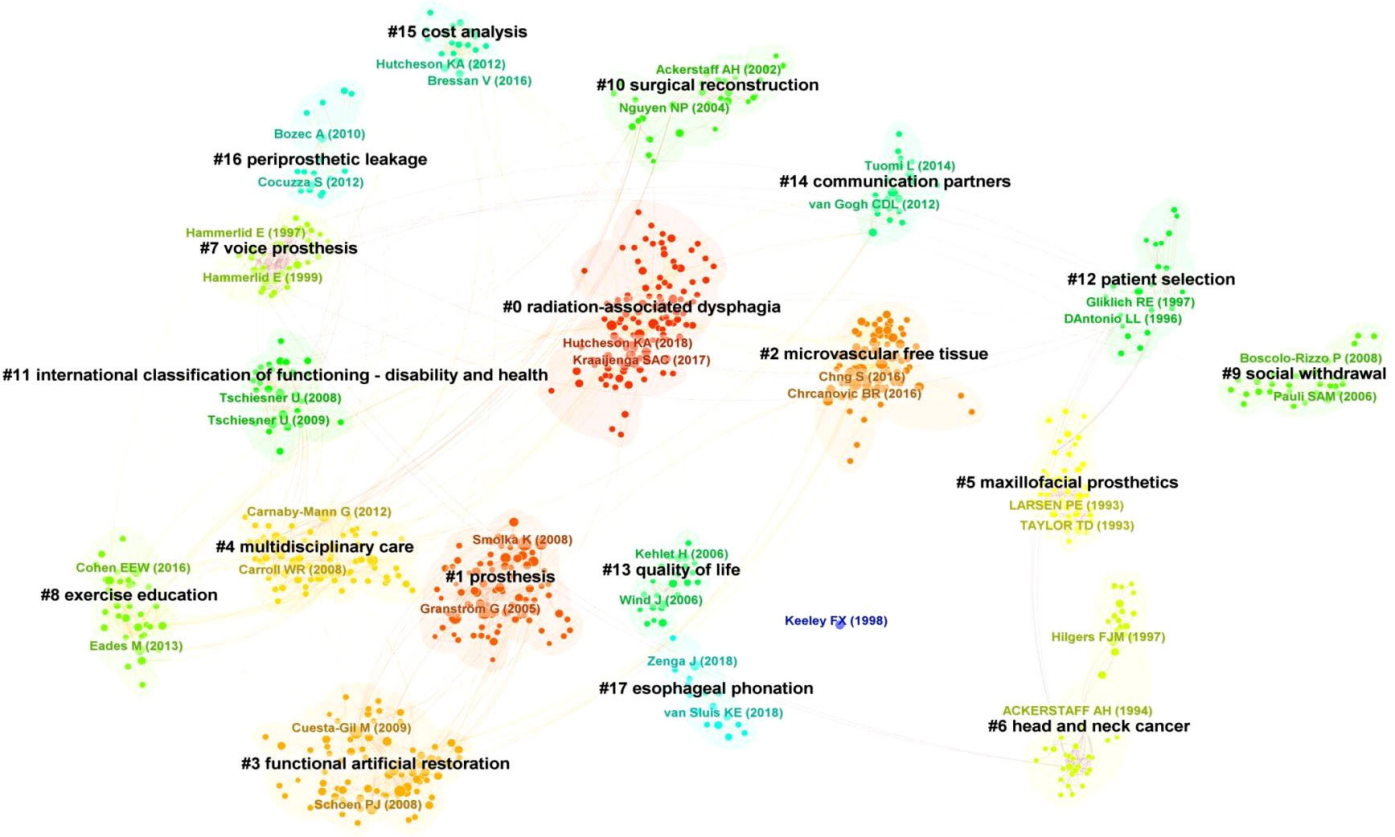
Analysis of literature co-citation clustering.

The co-citation analysis divided the literature in the field of head and neck cancer rehabilitation into 18 clusters (cluster numbering starts from 0) (**Figure 10**), namely #0 radiation-associated dysphagia (2017), #1 prosthesis (2004), #2 microvascular free tissue (2017), #3 functional artificial restoration (2011), #4 multidisciplinary care (2011), #5 maxillofacial prosthetics (1995), #6 head and neck cancer (1995), #7 voice prosthesis (1997), #8exercise education (1997), #9 social withdrawal (2014), #10 surgical reconstruction (2006), #11 international classification of functioning-disability and health (2003), #12 patient selection (2008), #13 quality of life (1998), #14 communication partners (2004), #15 cost analysis (2013), #16 periprosthetic leakage (2011), #17 esophageal phonation (2019). Among them, clusters 0, 9, 11 are related to the harms and complications of surgery, and research on the physical level precedes social function and psychology. Cluster 1, 2, 3, 4, 5, 7, 8, 10, 14 are closely related to the rehabilitation measures of head and neck cancer. Cluster 1, 2, 5, 10 are mainly solved by surgical means, while cluster 3, 4, 7, 8, 14 are achieved by postoperative rehabilitation through various means, such as auxiliary equipment, repeated functional exercises, social support, and psychological and behavioral therapy. In this field, due to the diversity of rehabilitation methods and the different social conditions of patients, cost analysis has also become an important content. In addition, the most advanced research is cluster 17 (2019) esophageal phonation.

## 4 Discussion

This study conducted a comprehensive bibliometric research in the field of head and neck cancer rehabilitation treatment. The characteristics of head and neck cancer-related rehabilitation research were studied from the perspectives of publication characteristics, countries, institutions, author collaboration, core journals, field development trends, research hotspots and so om. Over the past 30 years, there have been 1869 articles related to this field, and the number of articles has steadily increased, indicating that researchers are paying more attention to this field. However, it is not evenly distributed around the world and is mainly concentrated in a few countries in North America, Europe, and a few countries in Asia. At the same time, international cooperation in North America and Europe is relatively frequent, while international cooperation among countries in other regions is relatively weak. There is a phenomenon that there is less cooperation between multiple institutions in publishing documents. Although there are institutions with centrality greater than 0.1, the number of publications is relatively low. Head And Neck-Journal For The Sciences And Specialties Of The Head And Neck has published the most articles and achieved the highest citations, making it an undeniable core journal. Although Cancer has only eight articles, its published articles have an important position and profound academic significance in the field.

It’s particularly noteworthy that despite the United States leading in total publications on head and neck cancer rehabilitation, no US researcher is in the top ten publication count, while most listed are European researchers. This discrepancy highlights intriguing dynamics in research production and dissemination across regions. In order to explore the reasons for the formation of this phenomenon, we first consider the difference in funding models that significantly influence the nature and volume of research. In the US, medical research funding typically comes from centralized federal sources like the National Institutes of Health (NIH), which provides targeted, competitive grants. This model could lead to high overall publication numbers but may not support individual prolificacy due to the project-specific nature of funding. In contrast, European researchers often benefit from various funding sources, including substantial support from European Union (EU) frameworks, which may allow for longer-term projects and potentially more publications per individual.

Additionally, the academic and healthcare systems also play significant roles. The US system’s emphasis on translational or clinical research that leads directly to therapeutic outcomes encourages broad, multidisciplinary collaborations, often diluting individual publication counts. Conversely, European systems might provide more incentives for individual publications, evident in structures that more directly reward personal academic contributions.

While providing a preliminary understanding, these points are based on general observations and require further investigation for a deeper insight. Specific cases may vary significantly, reflecting researchers’ diverse academic and cultural contexts. Confirming these hypotheses would entail detailed interviews and analyses of particular research teams and their operational environments across both regions. These discussions not only shed light on the distinct patterns observed but also underscore the complex, multifaceted nature of global academic contributions and the myriad factors influencing them.

In addition to analyzing and discussing the results of this study, we also conducted a critical review to better illustrate the topic of this research. In the field of head and neck cancer rehabilitation treatment, research not only focuses on the influences of pathological types, anatomical locations, and surgical methods for rehabilitation, but also focuses on reconstruction surgery methods, prostheses, and related topics. It can also be seen that the field of research has shifted from focusing solely on survival rates, individual dysfunction, to patients’ overall physical functions, social functions, quality of life, and mental health. In terms of research trends, before 2008, the field developed slowly, with average bursts period of individual keywords experiencing lasting 10.6 years, indicating a slow rate of research breakthroughs and lower scholarly activity. After 2008, the time period was significantly shortened, with individual keywords lasting only 4.9 years, which means the research focus has been particularly short-lived, with scholars frequently changing their research topics. In addition, research now places greater emphasis on the level of clinical evidence. Nowadays, the rehabilitation treatment of non-surgical patients, rehabilitation cost analysis, and esophageal voice have also been widely studied.

Furthermore, as revealed by our co-citation analysis. Initially, research in the mid-1990s, as captured in the “Maxillofacial Prosthetics” cluster, concentrated on facial reconstruction, particularly involving the jaw and facial bones. This early focus aimed at restoring the basic contours and functionalities of the face, which are critical not only for patients’ physical appearance but also for essential functions such as chewing and speaking.

As the field progressed, by the late 1990s, the emphasis shifted towards enhancing patients’ quality of life through the restoration of voice functions, evident in the “Voice Prosthesis” cluster. This transition underscores the evolving rehabilitation needs, highlighting innovations in prosthetics that aid vocalization — a crucial aspect for patients who have lost their voice due to cancer treatments.

By 2004, the scope of research in prosthetics had broadened significantly, reflecting in the “Prosthesis” cluster. This period saw advancements in microsurgical techniques and the introduction of diverse types of prosthetics, such as those used for the temporomandibular joint. This expansion is indicative of a trend towards more specialized and refined rehabilitation needs, moving from basic functional restoration to detailed and comprehensive reconstructions that cater to various aspects of patient recovery. These phases of development in prosthetic research not only illustrate the technological advancements but also the shifting focus of rehabilitation efforts, which have progressively aimed to address more complex and nuanced patient needs.

Rehabilitation treatment for head and neck cancer is still flourishing. Therefore, we need more research and continuous observation on this topic to see whether research institutions and researchers in different countries will have a more in-depth and cutting-edge view on such topics. This means that despite these encouraging results, there are still problems in this study. There are at least two limitations to the study’s results. First of all, due to the relatively small amount of literature available in this field, the results of bibliometric analysis are limited and may not be able to fully describe all aspects of the research field. In addition, due to software design, compared with the older studies, it is difficult to find newly published high-level literature among the results of visual analysis. In the future, we will continue to improve these defects to approach the accuracy of trend forecasts.

## 5 Limitation

While our study provides comprehensive insights into the rehabilitation of head and neck cancer over the past 30 years, it is important to acknowledge certain methodological constraints. One significant limitation arises from our reliance on citation counts as a primary metric for evaluating the relevance and impact of the literature reviewed. This approach may not fully capture the immediate quality and innovativeness of newly published research, as these articles have had limited time to accumulate citations. To mitigate this, we considered additional metrics such as the impact factors of the journals, the credibility of the authorship teams, and the substantive content of the articles themselves. However, these measures involve subjective assessments and are not as easily quantifiable as citation counts, leading to potential biases and inconsistencies in evaluating recent publications.

Despite these limitations, our extensive review spanning three decades and incorporating 1869 documents provides a robust foundation for understanding long-term trends and shifts in the field. For future updates and to complement this study, we aim to conduct a focused review that will specifically target recent publications using a more qualitative approach to assess their contributions to the field. This will help in keeping the research community abreast of the latest developments and innovative practices in the rehabilitation of head and neck cancer.

## 6 Conclusion

In the past three decades, the number of publications in the field of head and neck cancer rehabilitation has increased year by year, and the average citation frequency has increased rapidly. Most important publications are published in high-impact journals. The United States has been a leading research country. A large number of research institutions and authors participated, and it was quite dispersed. This field shows a trend of cross-disciplinary development, including rehabilitation, surgery, oncology, materials science, psychology, social science, economics, etc. Future directions for further development include the continuous innovation of rehabilitation methods, seeking higher-level clinical evidence, more cost-effective rehabilitation methods, esophageal voice, patient mental health and quality of life. We also recognize the need for an integrated global approach to foster collaborative efforts and leverage diverse academic and clinical expertise, aiming to address disparities in publication output and contribute to the advancement of universally accessible rehabilitation methods.

## Data availability statement

The original contributions presented in the study are included in the article/supplementary material. Further inquiries can be directed to the corresponding author.

## Author contributions

BZ: Validation, Visualization, Writing – original draft. JC: Formal analysis, Supervision, Writing – review & editing. DL: Methodology, Supervision, Writing – review & editing. KD: Conceptualization, Methodology, Writing – original draft.
